# Facilitating bias in cost-effectiveness analysis: CHEERS 2022 and the creation of assumption-driven imaginary value claims in health technology assessment

**DOI:** 10.12688/f1000research.123709.1

**Published:** 2022-09-02

**Authors:** Paul Langley

**Affiliations:** 1 Adjunct Professor, College of Pharmacy, University of Minnesota, MINNEAPOLIS, MN, 55455, USA

**Keywords:** CHEERS 2022, facilitating bias, imaginary claims, model redundancy, analytical dead end

## Abstract

The current standards for health technology cost-effectiveness assessment rest on the creation of lifetime assumption-driven modeled simulations for imaginary pricing and consequent patient access recommendations. A recent *BMJ* paper reports a detailed assessment of 8,192 cost-effectiveness analyses, concluding that industry-sponsored modeled claims were more likely to publish incremental cost-effectiveness ratios (ICERs) below a USD 50,000 threshold than non-industry sponsored studies, supporting the claim that the product was cost-effective. This is unsurprising; indeed, the opposite can occur with a modeled claim deliberately resulting in ICER is excess of USD 50,000. This methodology is well entrenched with the recently published Consolidated Health Economic Evaluation Reporting Standards (CHEERS) 2022 guidance for creating imaginary cost-effectiveness modeled claims ensuring the opportunity for deliberately manipulated cost-effectiveness claims. This overlooks limitations imposed by fundamental measurement, rendering assumption-driven simulations redundant analytical exercises. Manipulation of ICERs and claims for cost-effectiveness are mathematically impossible; including cost-utility thresholds, because the preference or utilities supporting creation of quality-of-life years (QALYs) are ordinal scores. Nevertheless, with the promotion of CHEERS 2022, the belief in imaginary modeled value claims is both facilitated and reinforced. With CHEERS 2022, there is a concerted effort, largely in self-interest, to perpetuate the current belief system. This is a self-defeating strategy. Rather than admitting to the deficiencies of assumption-driven simulated imaginary claims, leaders are maintaining that health system decision makers can ignore standards of normal science and fundamental measurement in value claims for pharmaceutical products. This disregard of standards that are commonplace in the sciences and mainstream social sciences perpetuates the opportunity for self-serving modeled claims; where models are a marketing vehicle leading to sponsored systematic bias in formulary submissions. This supports the need for a NEW START paradigm for health technology assessment, focusing on evaluable single-attribute value claims, meeting the required standards for normal science and fundamental measurement.

## Introduction

A recent commentary in *F1000Research* made the case for the rejection of the current standards, the belief system or meme, in health technology assessment in favor of a NEW START paradigm that meets both the standards for normal science and fundamental measurement. ^1^ Value claims in normal science are required to be credible, evaluable and replicable; while fundamental measurement requires credible value claims to have interval or ratio measurement properties. The NEW START paradigm for value claims in health technology assessment requires the claim to be for a single attribute, to be unidimensional and to allow empirical evaluation within a meaningful and short timeframe. The NEW START rejects claims for cost-effectiveness as these are composite measures, with the claim resting on assumption driven, modeled lifetime simulations to produce outcomes that are entirely imaginary, putting to one side standards for normal science and measurement. The current health technology framework fails the demarcation test; it is pseudoscience. ^2^

Assumptions regarding model structure and parameter values are the core of the current health technology assessment (HTA) meme. The focus is on lifetime models that are intended to capture speculative approximate information for therapy response over the natural course of a disease. The models are not designed to create evaluable outcomes, but to simply ‘inform’ health system decision makers with simulated approximate claims; although, it is not clear what the approximate relates to. ^3^ More crucial, however, is the fact that as there is no basis in logic for believing claims from the past to support assumptions that will hold, even approximately, in the future; there can be any number of competing non-evaluable model claims. It is not a question of model transparency and justifying choice of assumption: the model is just one of a possible multitude of models each based on assumptions drawn from the literature and completed pivotal clinical trials; there is no justification for one set of assumptions than any other. Assumptions, it might be added, which are in many cases flimsy, based upon one or two studies and not a possible consensus in the literature.

If value claims for competing products are based on assumption, then there is the obvious incentive, in a competitive environment where formulary listing at a preferred price can have significant financial implications for industry, to compose modeled claims for cost-effectiveness which support the preferred price and formulary placement of the product. This brings in the question of bias and whether or not there is substantive evidence to support claims for manufacturers ‘gaming’ the system to create favorable cost-effectiveness claims, notably for drugs. The possible presence of bias, and the encouragement of guidance for imaginary claims as with CHEERS 2022 does nothing to alleviate the suspicions of formulary committees and health decision makers that they are dealing with a house of cards.

The purpose of this brief commentary is to make the case that, by continuing to support the current health technology assessment meme, bias is inevitable. Irrespective of efforts made to police the system with requirements for greater transparency and training for formulary assessors, if a modeled value claim is required to support a specific claim for cost-effectiveness, it will be constructed. Questions of bias will be easily deflected in justification for model structure and data inputs. Proposing guidelines for submitting imaginary modeled claims for cost-effectiveness, exemplified by CHEERS 2022 with its associated checklist will do no more than facilitate such endeavors; ^4^ justifying a continuing belief in model claims rather than rejecting a technology assessment meme which is manifestly deficient, fatally flawed and failing the demarcation test between science and pseudoscience.

## The McMaster–Tufts study

A recent evaluation presented in the *BMJ*, makes the claim that, in the universe of modeled cost-effectiveness claims, bias is pervasive in industry-sponsored studies. ^5^ This is, of course, an unexceptional conclusion as there is a wealth of evidence to support claims for bias where industry-funded cost-effectiveness claims are likely to report favorable results to the sponsor are readily published in leading journals. ^6^^,^^7^^,^^8^^,^^9^ The focus of the *BMJ* study is the Tufts University Cost-Effectiveness Analysis (CEA) Registry and the reporting of claims for studies published between 1976 and 2021; the majority in the last 10 years. ^10^ The study identified 8,192 studies, of which 46.5% were for drugs. A range of study categories were identified, but aggregated to industry sponsored as opposed to non-industry. Studies were categorized in terms of disease and methodological characteristics, with incremental cost-effectiveness ratios defined in terms of three cost-per-QALY thresholds: USD 50,000, USD 100,000 and USD 150,000. A total of 2,437 (29.7%) of CEAs were sponsored by industry. Of these, 90.3% were model based (compared with 89.2% for non-industry studies) with 78.7% of industry sponsored CEAs having an ICER below USD 50,000 *versus* 65.4% for non-industry sponsored; a gap of 13.3% of the industry sponsored. This does not mean, of course, that non-industry-sponsored CEA models were not designed to produce claims also below a selected imaginary cost-per-QALY threshold; perhaps bias, the deliberate choice of specific assumptions is the construction of modeled approximate-information value claims is more prevalent than might be thought.

In the base case logistic regression with 8,192 CEAs, industry-sponsored claims were more likely to conclude that the intervention was cost-effective than the comparator with a threshold of USD 50,000.; an adjusted odds ratio of 2.06 and a 95% CI of 1.82 to 2.33. Similar results were reported for the other two thresholds. In terms of magnitude, industry-sponsored CEAs’ ICER was 33% lower than non-industry sponsored (95% CI −40% to −26%). The analysis suggested that the industry-sponsored bias was systemic, existing across a wide range of disease and study designs. A further subgroup analysis found that CEAs for drugs accounted for almost three-quarters of industry-sponsored studies compared with just over a third for non-industry-sponsored studies, with one of the largest sponsorship biases. Perhaps not surprisingly, the analysis found least bias among trial-based as opposed to modeled studies.

As assumption-driven simulation models have no external reference point (apart from other studies), the ability to compose value claims for cost-effectiveness are obvious. Challenging assumptions drawn from the existing literature to populate non-evaluable claims for an unknown (and unknowable) future are clearly a fruitless activity. Any set of assumptions can be defended unless, as with the National Institute for Health and Care Excellence (NICE), there is a regiment of academic-assumption assessors that can pronounce on submitted model claims. It is not clear why anyone would want to engage in such an activity unless one is committed to non-evaluable imaginary claims as a sure basis for formulary submissions, in line with the promotion of imaginary approximate information by the International Society for Pharmacoeconomics and Outcomes Research (ISPOR). ^3^

## Accepting pseudoscience

To accept the argument for industry bias in composing modeled cost-effectiveness claims seems somewhat paradoxical when the basis on which the assessment has been undertaken accepts a meme that fails the standards for normal science and measurement. The Tufts University CEA database pays no attention, or is ignorant of, the standards for fundamental measurement. Multiattribute utility or preference scores ( *e.g.*, EQ-5D-3L/5L) are presented as a fee-based ‘help-yourself’ emporium but with no understanding that these are ordinal scores. As such, they cannot, as ordinal scores, support the universally favored yet mathematically impossible QALY and the various economic models. ^11^ This means that the many applications by ICER to create QALY claims are also mathematically impossible as are cost per-QALY thresholds. The criteria for assessing industry bias; the extent to which industry ICERs squeak under thresholds is an impossible measure. In this important sense, the entire *BMJ* analysis is redundant. Added to this is the failure to recognize that the entire modelling meme is unsustainable; non-empirically evaluable value claims are simply a chimera. Yet they continue to be endorsed and published by manufacturers and academic groups.

If protagonists are to argue for industry bias, then they must embrace the pseudoscientific basis of approximate-information modelled outcomes; including the notion of ordinal cost-effectiveness as a believable metric. If the modeling is rejected, then cost-effectiveness claims disappear, together with the measurement of bias. The ready availability through the Tufts’ database for access to ordinal preferences or utilities makes, unfortunately, the construction of imaginary claims that much easier. The application of the Tufts’ data is illegitimate. Certainly, it is possible to apply a detailed regression analysis, but that presumes that all data elements in the model meet required measurement standards; the Tufts’ data certainly do not.

## The price of failure

While it is one thing to point out that under the current approximate-information, health technology assessment meme it is possible to have a range of competing assumption-driven models, it is another to attempt to provide a *prima facie* case that for industry it is a question of how to game the system to generate cost-effectiveness claims that are deliberately constructed to meet favorable cost-per-QALY thresholds. A process for modeling and choice of assumptions that is made more straightforward once cost-per-QALY thresholds are established and the objective is to create a cost-effectiveness case that deliberately yields a value just short of the threshold value.

In an important sense, the approximate-information assumption-driven simulation meme is hoist with its own petard. A standard for health technology assessment for 30 years; it is also a meme that is driven by non-evaluable value claims; biased or otherwise. The fact that the standards of normal science for credible, evaluable and replicable claims are rejected merely opens Pandora’s Box. There are not the resources and skills, with a failure in training and education, to assess (and reject) these approximate-information models. As long as these standards are accepted by health-system decision makers and journal editors, as witnessed by the endorsements of the CHEERS 2022 guidance, the opportunity and rewards from ‘threshold’ composition will continue. This absence of resources and skills, it might be added, is an added incentive to create favorable CEA claims. It is all very well for agencies, such as NICE in the UK and the Pharmaceutical Benefits Advisory Committee (PBAC) in Australia, to establish academic-review centers, staffed by those who have devoted their professional lives to challenging and modifying assumptions in imaginary lifetime models; but this is only possible in the real world if the resources are available to journal editors and if there is a willingness to engage with industry in what is best seen as a pointless activity.

Of course, even if a concerted effort were made in graduate programs, health systems and even industry to provide a more coherent basis for developing and evaluating model claims, the exercise would be a waste of effort. It is not, to emphasize, the problem of systemic bias, but of a more fundamental objection to the belief in modeled imaginary claims. The assessment meme fails the standards of normal science and measurement and should not be subject to an assessment of bias in the first place. ^1^ The *BMJ* findings are of interest, and indicative of the ease with which models potentially can be deliberately manipulated, but of limited interest given the more pressing issue of required standards for formulary submissions and the discovery of new facts for therapeutic benefit.

## Implications for ICER-Modelled claims

Although the *BMJ* analysis does not identify industry *versus* non-industry CEA studies by country, the analysis has some major implications for the modeling by ICER in the USA. Previous commentaries have pointed out that the ICER embrace of assumption-driven simulated model claims defies the standards of normal science and fundamental measurement. ^12^^,^^13^ Yet, with the continued acceptance of the approximate-information meme, ICER through its stable of academic consultants, keeps producing imaginary modeled recommendations for pricing, with threshold cost-per-QALY cutoffs, and access to the selected drugs. The *BMJ* study points quite clearly to the futility of this approach in that ICER’s modeling produces only one or a selection of many possible imaginary value claims for cost-effectiveness and pricing to achieve threshold cutoffs. This is made clear by the commitment by many manufacturers to tailor their modeling to meet threshold constraints. This is not to justify, as in the case of the *BMJ* study results, a commitment to minimize the opportunities for bias through review or even the support for ‘blinded’ competing models; this is impossible and unnecessary given the deficiencies of approximate-information modeling. What the *BMJ* study makes clear is that once an approximate-information meme is the vehicle for cost-effectiveness claims, health system decision makers should reject those claims. Virtually any value claim, engineered or otherwise, must be rejected.

The existence of systematic bias is a salutary reminder of how futile is this approach to value claims; it might be taken one step further to argue that bias is inevitable in any choice of assumptions. The problem with this argument is that the presence and extent of possible bias is unknown as there is no reference point for judging assumptions about an unknown future. Hopefully, the *BMJ* study will be seen in the US as further evidence for rejecting assumption-driven cost-per-QALY simulations where one imaginary study is as good or as bad as another, with no value at all to health system decision makers. There should be no encouragement given to industry to compete with ICER in the imaginary cost-effectiveness claims stakes.

But this encouragement is present with CHEERS 2022. The door is not only open, but a guidance supported pathway for those wishing to compose their own selective assumption-driven imaginary simulation to support imaginary claims for cost-effectiveness; a guidance that in particular with its support of the QALY, fails to recognize that it is a mathematically impossible construct.

## New start paradigm

As detailed in the previous commentary ^1^ credible value claims for any pharmaceutical product or device must be presented as protocol driven single attributes with ratio or interval measurement properties; blanket claims such as those favored by ICER and CHEERS 2022 for imaginary modeled cost-effectiveness fail these requirements. NEW START rejects completely the existing assumption-driven approximate-information meme. ^1^ Rather, it starts from the premise that if technology assessment in health care is to be meaningful it must meet the standards for normal science and measurement. Value claims must be single attribute and target patient populations or specific disease states. All claims must be for unidimensional attributes with interval or ratio measurement properties irrespective of whether they are for clinical outcomes, patient reported outcomes (PROs) or drug and resource utilization. Composite or multiattribute generic claims are unacceptable, as will be disease specific PRO claims that fail to meet Rasch or modern measurement standards. This means that the bulk of PRO claims must be rejected as they only produce ordinal scores.

NEW START also minimizes the opportunities for gaming the system; composing models that favor a sponsor. This follows from the requirement that all claims must be supported by an evaluation protocol to detail how the claim is to be evaluated and reported in a meaningful time frame. If all value claims are required to be empirically evaluable there is a firm bass for rejection in an ongoing process of conjecture and refutation; a process that is alien to the approximate-information meme. The fact that NEW START value claims are only provisional means that there is no possibility of squeaking under the radar with imaginary, purpose-built modeled value claims and imaginary thresholds.

## Conclusions

The uncomfortable truth is that for many participants there is no incentive to reject the current approximate-information meme. Not only is it well entrenched, but industry as the prime mover with deep pockets has no incentive to change; it is an easy option to build a one-off modeled imaginary value claim for cost-effectiveness rather than engage in a long-term research program of therapy response for the target patient population or disease state. Over the past 30 years thousands of analysts have embraced assumption-driven imaginary claims; careers have been built on it and professional associations have unquestioningly adopted it. There are too many with too much to lose.

Against this commitment (or inertia) is the growing realization that the meme for technology assessment is not only built on sand, with a complete disregard for the standards of normal science and fundamental measurement, but with a real and demonstrated ability of the meme to facilitate systematic bias. This is not only an implication for study designs and diseases in industry sponsored assumption-driven modeled cost-effectiveness threshold value claims, but more widely in the composition of any assumption-driven imaginary simulation. Sponsored modeling can always support, if published, the sponsor’s product. After all, that is what many believe consultants are for in health technology assessment. The meme, in this very real sense, despite its acceptance, is self-defeating; there must always be a suspicion that there are elements in the model that, deliberate or not, are questionable and impact outcome claims. Embracing CHEERS 2022 is no guarantee that the pursuit of self-serving assumption-driven modeled imaginary claims will not continue; indeed CHEERS 2022 encourages it in its support for the submission of modeled claims to journals; journals which have limited resources and skills seriously to challenge and reassess the structure and specific assumptions driving the mode, even though it fails the standards of normal science. Indeed, it is not just a self-defeating analytical framework but one that from the very start has promoted the support for non-evaluable claims. A position unique among the mainstream social sciences such as economics.

Formulary committees and other health decision players have no reason to believe, let alone support, assumption-driven modeled imaginary claims for cost-effectiveness. Instead, the option is there with the NEW START formulary submission package which not only meets the required standards but in its emphasis on evaluable protocol driven, single attribute value claims limit the opportunities for favorable constructs. Value claims for patient reported outcomes such as utility scores and quality of life must conform to the same standards for claims proposed and evaluated in randomized clinical trials. The approximate-information CHEERS meme has long passed its use by date. If, of course, we were serious about imaginary approximate information then the ideal solution would be for health system decision makers to make clear that imaginary claims are not relevant when judged by the standards of normal science and have no role in formulary decision making. Unfortunately, given the endorsement by leading journals of the CHEERS 2022 guidance, we cannot rely on journal editors to entertain accepting only value claims proposals that meet the standards of normal science; the opportunity and incentive to publish biased yet imaginary modeled value claims will remain as a marketing standby.

## Data availability

There are no data associated with this article.
