# Supplementary material for: Facilitating bias in cost-effectiveness analysis: CHEERS 2022 and the creation of assumption-driven imaginary value claims in health technology assessment
Source: F1000Res. 2022 Sep 2;11:993. [Version 1] doi: 10.12688/f1000research.123709.1 (PMC9525991; doi:10.12688/f1000research.123709.1)
Supplement: Peer review history

# Peer review: Reviewer response for version 1

**DOI:** 10.5256/f1000research.135842.r149391

**Published:** 2022-10-03

**Authors:** Jonathan Belsey

**Affiliations:** 1 JB Medical Ltd, Sudbury, UK

The author presents a cogently argued rationale for the rejection of the current paradigm of cost-utility assessment based on the use of QALYs derived from questionnaire-derived utility assessments. There are significant methodological limitations in the derivation of utility estimates, which are further compounded by the use of inappropriate statistical approaches to manipulation of the consequent QALY estimates.

 The author has previously published on this issue and I am in agreement with him with regards to the invalidity of the existing methodology. However, translating this understanding into a global willingness to shift the paradigm to a more objective and reproduceable metric is a massive challenge and I think it unlikely that this will happen within the span of my remaining career. There are huge industries invested in the current approach. Health Technology Appraisal bodies, commercial health economics consultancies, HEOR departments within pharma companies as well as academia have all followed this path for the past 25 years. Regardless of whether the individuals within this loosely affiliated structure of organisations recognise the truth of what the author describes, the process of change is too threatening and destabilising to allow them to go down this path comfortably.

 I would like to think that those of us in the field of Health Economics are scientists and that we are capable of acknowledging the shortcomings of the current approach and instituting change. I suspect, however, that we are too vested in the accepted vision to make this move.

 I hope that the author can take on the role of the outspoken child in "The Emperor's New Clothes". I admire him for his efforts, which are well-argued and compelling. I fear, though, that he will simply be trampled in the rush to admire the grand Imperial vestments!

Is the topic of the opinion article discussed accurately in the context of the current literature?

Yes

Are arguments sufficiently supported by evidence from the published literature?

Yes

Are all factual statements correct and adequately supported by citations?

Yes

Are the conclusions drawn balanced and justified on the basis of the presented arguments?

Yes

Reviewer Expertise:

Health economics and Health Technology Appraisal (UK)

I confirm that I have read this submission and believe that I have an appropriate level of expertise to confirm that it is of an acceptable scientific standard.

# Peer review: Reviewer response for version 1

**DOI:** 10.5256/f1000research.135842.r149390

**Published:** 2022-09-30

**Authors:** Ariel Beresniak

**Affiliations:** 1 Data Mining International, Geneva, Switzerland

The article is enriching the international controversy of using the QALY indicator in formulary decision making, leading to a number of pharma sponsored modelling studies published in the scientific literature.

 The author denounces the inconsistency of the QALY indicator, mainly because it is an ordinal metric inadequately used as a ratio (cardinal) metric for calculating ICER. This is not the only methodological limitation of the QALY indicator which is derived from a multiplicative model based on other very fragile assumptions, which directly influence the model results and the ensuing decisions.

 However, it is not certain that the article would be fully understandable by non-specialists in outcome research, which would justify a better explanation of the basic concept in an educational way.

 For example, the literature confusion between cost-effectiveness and cost-utility should be explained in the article as cost per QALY models are theoretically cost-utility models often presented as pseudo cost-effectiveness models. This clarification would be very helpful for most of the readers.

 The use of additional examples could also improve the understandability of the article. For example the fact that divergent ICER could be derived from the same dataset, depending on the sponsor perspective would have been interesting to discuss in this opinion article.

 The comments about the CHEERS guidelines endorsed by a number of scientific journals without any assessment of their validation are relevant because such guidelines support the use of QALY, which is adequately considered aby the author as pseudo sciences but contribute to a very profitable economic business between academic and private consultants financed by the industry. A short additional discussion about how scientific journals are encouraging flawed methodologies would be appropriate.

Is the topic of the opinion article discussed accurately in the context of the current literature?

Yes

Are arguments sufficiently supported by evidence from the published literature?

Yes

Are all factual statements correct and adequately supported by citations?

Yes

Are the conclusions drawn balanced and justified on the basis of the presented arguments?

Yes

Reviewer Expertise:

Public Health, Health Economics, Outcome Research

I confirm that I have read this submission and believe that I have an appropriate level of expertise to confirm that it is of an acceptable scientific standard.

# Peer review: Reviewer response for version 1

**DOI:** 10.5256/f1000research.135842.r149389

**Published:** 2022-09-20

**Authors:** Uwe Albrecht

**Affiliations:** 1 Mediconomics, Hanover, Germany

The article is well written and gives insight into a situation that needs to be addressed if the decision-making in the core markets for pharmaceutical products regarding reimbursement and availability is intended to be based on reliable facts avoiding fiction.

 Science develops. As medicine is not a typical natural science, we have become used to relying on various methodologies to explain the best possible utilization. However, even Einstein´s theories – based on fundamental principles of science – experienced further exact modelling by Hawking many years later. Why is there reluctance to change to a New Start in evaluating medicines? We want to make the best treatment available and to keep a close eye on associated costs. Therefore, the elements are first to prove efficacy and safety with fundamental physical measurements (if possible) and in conjunction with the fact that the desired need of a patient is fulfilled by the treatment. And this is key: Costs must be outweighed against desired treatment outcomes.

 The underlying models must be a dedicated developed piece of science and generate data that are the desired reflections of science. As the author rightly mentions, it has become normality to use available tools and is often regarded as a standard by the institution and its staff. The decision-makers have guidance papers, but is there an education looking into meaningful assessment? From 35 years in the triangle of clinical research, regulatory affairs, and marketing, I can report that each party has its tools and views. Moreover, of course, marketing wishes to place a product right in the therapeutic niche to generate payback on development costs. This is not wrong – it guarantees the survival of the most vital part of our system. But it may explain why models a geared towards desired proof of outcome. A hen and egg problem. Why not first determine what is required to make the product the best alternative to fulfil the needs of a patient group? Then make a product that fits and determine that the needs are fulfilled in a proper scientific sense. If this is transparent, less complicated modelling will be required. Back to physics, there are mathematical approaches to explain complex situations far beyond the horizon of a scientist from other disciplines, and only sometimes critical parts of excellent science cannot be explained in depth. However, medicine and outcomes are not reflecting on particles being transiently there and then partly in a black hole. The opposite is accurate, and we have the ideal situation to re-think and be encouraged to make a NewStart, thereby taking care of our resources. The article should be regarded as a wake-up call to explore new avenues with a clear understanding of what we need. The vaccines to fight the pandemic have shown that there is a lack of need fulfilment. Did we want a vaccination that may or may not have the power to avoid the disease in its indication and have the healthcare systems paying for it in full? Room for improvement is there, and likewise, for other medicines under development.

Is the topic of the opinion article discussed accurately in the context of the current literature?

Yes

Are arguments sufficiently supported by evidence from the published literature?

Yes

Are all factual statements correct and adequately supported by citations?

Partly

Are the conclusions drawn balanced and justified on the basis of the presented arguments?

Yes

Reviewer Expertise:

NA

I confirm that I have read this submission and believe that I have an appropriate level of expertise to confirm that it is of an acceptable scientific standard.
